# A missense mutation in the *KCNE4* gene is not predictive of equine anhidrosis

**DOI:** 10.1111/age.70004

**Published:** 2025-02-15

**Authors:** Lexie van der Graaf, Wesley Leigh, Tomasz Szmatoła, Kelsey Roberts, Stephanie Ryan, Briana Brown, Samantha Van Buren, Carrie J. Finno, Jessica L. Petersen

**Affiliations:** ^1^ Department of Population Health and Reproduction University of California‐Davis, School of Veterinary Medicine Davis California USA; ^2^ Department of Basic Sciences University of Agriculture in Krakow Kraków Poland; ^3^ Department of Animal Science University of Nebraska‐Lincoln Lincoln Nebraska USA

**Keywords:** genetic testing, horse, sweat, validation

## Abstract

Anhidrosis is defined as a decreased or absent ability to sweat in response to heat and exercise. In horses, this condition can increase the risk of life‐threatening hyperthermia. A prior study has suggested that equine anhidrosis is associated with a missense variant (rs68643109) in the Potassium Voltage‐Gated Channel Subfamily E Regulatory Subunit 4 (*KCNE4*) gene. This project aimed to validate this association in a population of well‐phenotyped horses and to determine the allele frequency of this variant in publicly available whole‐genome sequence data. Fifty horses within the University of California Davis Center for Equine Health herd were evaluated for anhidrosis using a series of intradermal terbutaline injections. From existing whole‐genome sequence data, the rs68643109 genotype of each horse was identified. When stimulated with terbutaline, all 50 horses produced sweat. All three genotypes at rs68643109 were present in this population of horses; the allele previously associated with anhidrosis (G) was present at a frequency of 0.72. No statistical difference in total sweat score was found (*p* = 0.31). In whole‐genome sequences from 820 other horses reported across three prior studies, the alternative (candidate) allele frequency was similarly high, ranging from 0.52 to 0.68. Since all 50 horses tested in our population produced sweat regardless of genotype, and the previously associated allele is present at a high frequency across datasets, these data fail to validate the missense variant within the *KCNE4* gene as causative of or contributing to equine anhidrosis.

## INTRODUCTION

Anhidrosis, the decreased or absent ability to sweat in response to heat, is typically observed in horses living in hot and humid climates; increased rates of occurrence are present in populations involved in strenuous exercise (Johnson et al., 2010). In Florida, prevalence rates of the condition have been reported as high as 6.2% (Mayhew & Ferguson, 1987) with increased occurrence in the warmer central and southern regions of the state (Johnson et al., 2010). The severity of anhidrosis varies, with some horses having a decreased ability to sweat and others lacking the ability to sweat entirely (Jenkinson et al., 2007). Signs of the condition include increased rectal temperature and limited or absent sweat in response to physical exertion and/or thermal stress (Mayhew & Ferguson, 1987). In more severe anhidrosis cases, affected individuals may display hair loss, skin inelasticity, anorexia, decreased water consumption, elevated peripheral temperature and a dry, rough coat (Evans et al., 1956; Mayhew & Ferguson, 1987). Additionally, affected horses have an increased resting respiratory rate, body temperature and pulse rate compared with unaffected horses in the same conditions (Hubert & Beadle, 2002). The loss of ability to sweat increases a horse's potential to become hyperthermic, which can result in collapse, convulsions and even death (Warner & Mayhew, 1983).

Horses’ primary heat loss mechanism is cutaneous evaporation from sweat, which allows for the regulation of body temperature and the countering of thermal stress (Hodgson et al., 1994). Since anhidrotic horses typically possess sweat glands with structural and functional abnormalities, this suggests that the condition is a result of mechanical failures within the glandular components of sweat production (Jenkinson et al., 1985). As the condition progresses, the sweat gland undergoes cytoplasmic vesicle loss and epithelial thinning without the dilation of intercellular spaces and myoepithelial contraction that occurs in non‐affected horses during sweat production (Jenkinson et al., 1985). In chronically anhidrotic horses, the visible effect on sweat glands becomes more apparent as basal laminae appear thickened, ductal and fundic epithelial cells thin, and glands are visibly surrounded by fibrous tissue (MacKay et al., 2015). Furthermore, it is also postulated that blockage of the sweat gland ducts owing to excess secretory product and/or swelling and malformation contributes to anhidrosis (Jenkinson et al., 2007).

Although the genetics of anhidrosis in horses is relatively unstudied, one recent publication attributed risk of equine anhidrosis to a missense variant in *KCNE4* (Patterson Rosa et al., 2021). *KNCE4* encodes for a potassium channel membrane protein that acts as an inhibitory *β* subunit to KCNQ1 channels (Grunnet et al., 2002). The implication of *KNCE4* was based upon a genome‐wide association including data from 670 000 single nucleotide polymorphisms and 200 horses (100 control, 100 affected); the classification of anhidrosis was based upon owner surveys. The authors then used whole‐genome sequence data from one horse with chronic anhidrosis and one control to suggest that the missense variant rs68643109 (NC_009149.3:g.11813731A>G) contributes to the incidence of anhidrosis. That study (Patterson Rosa et al., 2021) led to the development of a commercial genetic test for the *KNCE4* variant that is interpreted to indicate a horse's risk of developing chronic anhidrosis. Although both *KCNE4* and *KCNQ1* are expressed in horse skin (Mansour et al., 2017), and the KCNQ1 channel is more prevalent in normal mice compared with sweat‐gland absent mouse models (Cui et al., 2016), information outlining the role of either *KNCE4* or *KCNQ1* in equine sweat production is currently lacking.

It is crucial to note that no committee or association currently assesses the quality of DNA tests available for animals (Finno & Bannasch, 2014). The false association of genetic variation with disease risk can have severe consequences for horses, owners and veterinarians, possibly resulting in incorrect diagnoses, unnecessary treatments, ill‐informed breeding decisions and incorrect husbandry practices. Given this, we aimed to test the validity of the claim that anhidrosis results from the missense variant in *KCNE4* (rs68643109) utilizing a quantitative intradermal terbutaline sweat test (QITST), which is the gold standard in clinical practice for the evaluation of equine anhidrosis. We hypothesized that this mutation is not associated with anhidrosis and horses possessing the alternative (risk) allele would sweat normally in response to the QITST.

## MATERIALS AND METHODS

### Animals

The University of California (UC) Davis Institutional Animal Care and Use Committee approved all animal procedures (Protocol 23 250), and the UC Davis Center for Equine Health owned all horses studied. A total of 50 horses underwent testing for the presence of anhidrosis. Females (36 mares) represented 72% of the test population, whereas males represented 28% (12 geldings, two stallions). Quarter Horses (*n* = 19, 38%) and Thoroughbreds (*n* = 11, 22%) comprised the majority of the study group. Breeds comprising the remaining sample included Paint (*n* = 4, 8%), Warmblood (*n* = 4, 8%), Standardbred (*n* = 3, 6%) and other (*n* = 9, 18%) (Table S1).

### Quantitative intradermal terbutaline sweat test

**FIGURE 1:**
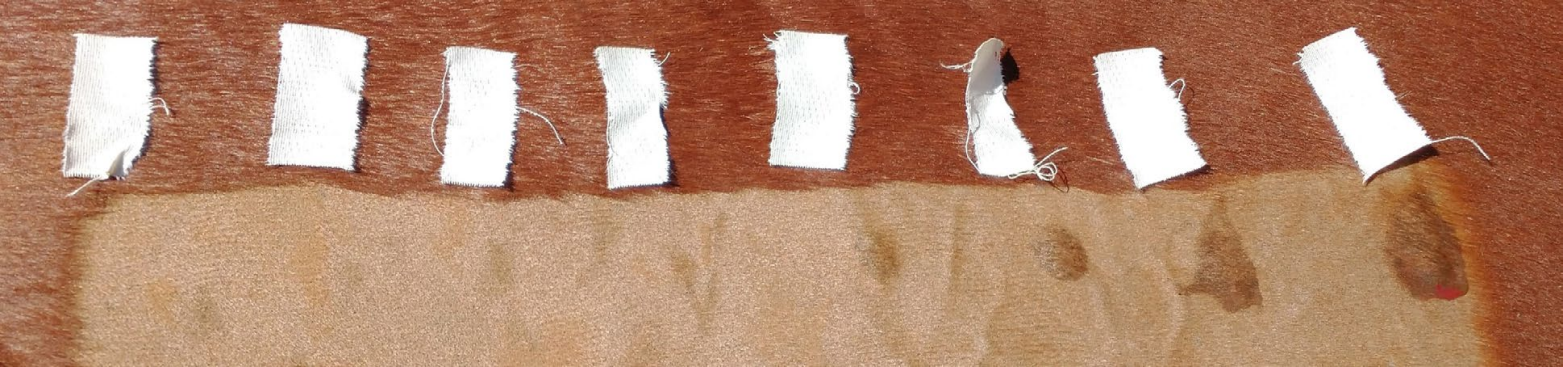
The quantitative intradermal terbutaline sweat test (QITST) of a study horse. The tape provides an approximate location of injection of the saline control (left) followed by serial dilutions of terbutaline sulfate (left to right: 10^−6^, 10^−5^, 10^−4^, 10^−3^, 10^−2^, 10^−1^, 10^0^ mg/L). This horse had no sweat (score of 0) for the control injection and terbutaline sulfate injections with concentrations of 10^−6^, 10^−5^, and 10^−4^ mg/L. A score of 1 was given for injections of 10^−3^ and 10^−2^ mg/L, and a score of 2 for the 10^−1^ and 10^0^ mg/L injections.

A quantitative intradermal terbutaline sweat test was administered to each horse, as previously described (MacKay, 2008). Briefly, a strip of hair, approximately 6–8 cm wide, was clipped parallel to the crest of the neck, approximately 5 cm below the dorsal margin. Starting near the poll, serial dilutions of 0.1 mL intradermal terbutaline sulfate in 0.9% saline were injected through a 25‐gage needle at eight sites, approximately 5 cm apart, as follows: 0 (control/saline), 10^−6^, 10^−5^, 10^−4^, 10^−3^, 10^−2^, 10^−1^ and 10^0^ mg/L (MacKay, 2008) (Figure 1). Thirty minutes after the administration of the final injection, the presence of sweat at each injection site was visually scored according to the following scale: 0 (no presence of sweat), 1 (sweat area visible but smaller than the size of a quarter), 2 (sweat area visible and larger than the size of a quarter) and 3 (excessive sweat response, usually in the form of a visible drip). In addition to considering the trait as binary, the sweat scores across all injection sites were summed, creating a total sweat score used to evaluate the sweat severity for each horse.

### Whole‐genome sequencing

Whole‐genome sequence data (~30× coverage) were available for the 50 horses as part of a large precision medicine initiative at UC Davis (Donnelly, 2022). Variant call format (.vcf) files were filtered using SNPSift (Cingolani et al., 2012) to obtain the genotypes for the putative causative variant; genotypes were validated by visualizing .bam files using the Integrated Genome Viewer (Robinson et al., 2011).

### Statistical analysis

Statistical analyses were used to evaluate the effect of genotype on sweat production in the test population. Normal distribution of the data was verified using a Kolmogorov–Smirnov test (Massey Jr, 1951) and a one‐way ANOVA conducted to determine if the total sweat score varied by genotype at position rs68643109. Results are reported as mean ± standard deviation, with *α* < 0.05 considered significant.

### Variant frequency in additional samples

To further evaluate the frequency of genotypes at the candidate locus, three large, publicly available whole‐genome sequence datasets were used, including a study of 185 US Thoroughbreds (Bailey et al., 2024), 101 Japanese Thoroughbreds (Tozaki et al., 2021) and 534 horses from 40 breeds (Durward‐Akhurst et al., 2021); see the Data Availability statement for accession information.

## RESULTS

All horses sweated during the QITST, with an average total sweat score of 6.82 (range 3–10) (Table S1). Thus, no horses were anhidrotic in this sample (*n* = 50).

In the 50 horses studied, the frequency of the previously associated allele was 0.72 (reference allele frequency 0.28). Individuals homozygous for the alternative allele (G/G, *n* = 18) had the highest average total sweat score (7.11; SD = 1.08), with heterozygous individuals (*n* = 27) having a mean total sweat score of 6.78 (SD = 1.65) and homozygous reference individuals (*n* = 5) a mean total sweat score of 6 (SD = 1.23) (Table 1, Figure S1). There was no significant effect of genotype on sweat score (*p* = 0.31).

**TABLE 1:** Mean sweat score for each injection and mean total score by genotype (A allele is reference, G is alternative). Standard deviation is given in parentheses. There was no difference in total sweat score by genotype (*p* = 0.31).

Genotype (*N*)	Saline	mg/L terbutaline sulfate							Total score
		10^−6^	10^−5^	10^−4^	10^−3^	10^−2^	10^−1^	10^0^	
A/A (5)	0	0	0	0.20 (0.45)	1 (0)	1.20 (0.45)	1.60 (0.55)	2 (0)	6 (1.22)
A/G (27)	0	0	0.07 (0.27)	0.56 (0.51)	0.93 (0.38)	1.26 (0.53)	1.81 (0.40)	2.15 (0.36)	6.78 (1.65)
G/G (18)	0	0	0.06 (0.24)	0.44 (0.51)	1 (0)	1.47 (0.51)	1.89 (0.32)	2.22 (0.43)	7.11 (1.08)

The variant allele frequency in the three large publicly available datasets was 0.524 in the US Thoroughbreds, 0.614 in the Japanese Thoroughbreds and 0.683 across the 534 horses of various breeds.

## DISCUSSION

In this study, we demonstrate that the missense variant in *KCNE4*, previously proposed to be a risk factor for equine anhidrosis, is not associated with sweat phenotype as determined by a QITST in our sample of 50 horses. In addition, the allele proposed to be a risk factor for anhidrosis by Patterson Rosa et al. (2021) was the major allele not only in this study sample but also in publicly available datasets that include 820 other horses across breeds. In one instance on their website describing the commercial test, the testing company lists the reference allele (A) as ‘mutant’ despite the publication naming the alternative allele as putatively causative. Nevertheless, the fact that all horses produced sweat and that total sweat score did not differ by genotype indicates that this locus should not be used in diagnostic testing for equine anhidrosis. Further research is needed to identify genetic markers for equine anhidrosis and to refine the current understanding of the disease's underlying mechanisms.

Study limitations include the relatively small sample size (only five horses had the reference (A/A) genotype), the lack of anhidrosis‐positive individuals and the environment where these horses were maintained (i.e. dry heat vs. humid). Recovery from anhidrosis can occur when horses are moved to cooler climates (Jenkinson et al., 1989). However, when the QITST procedure was used on a cohort of horses in Florida, there was no impact of environmental conditions or time of year on the QITST results (MacKay, 2008). Despite the lack of anhidrotic horses in our test population, the high frequency of the variant allele refutes its association with this condition.

At the time of this study, our findings have implications for the commercially offered genetic test for equine anhidrosis, which is reliant on this marker. These data provide compelling evidence that refute the previously proposed association between a missense variant in *KCNE4* and equine anhidrosis. Results from the current diagnostic test should not be utilized in management, care or breeding decisions. This outcome underscores the importance of rigorous validation in genetic studies, further highlighting the necessity of thorough vetting of commercial tests before they are made public. As no governing body or association examines the validity of genetic marker tests for animals, this is an example of how unregulated products have the potential to negatively affect patient care and treatment strategies. Advancing the understanding of the genetic components of equine anhidrosis with additional studies and proper validation of the results could lead to improved diagnostic accuracy, welfare and management of affected individuals.

## FUNDING INFORMATION

This project was supported by the UC Davis Center for Equine Health with funds provided by the State of California pari‐mutuel fund and contributions by private donors.

## CONFLICT OF INTEREST STATEMENT

None declared.

## Supporting information


Figure S1.

Table S1.

## Data Availability

Whole‐genome sequence data of the 50 study horses can be accessed using the National Center for Biotechnology Information Sequence Read Archive (NCBO SRA) accessions PRJNA841639, PRJNA553581 and PRJNA601992. Accessions of data utilized to quantify allele frequency across a larger sample include: Bioproject PRJNA993255 and accessions SRR19364580, SRR19364583, SRR19364585, SRR19364586, SRR19364587, SRR19364589, SRR19364593, SRR19364602, SRR19364605, SRR19364613, SRR19364619, SRR19364621, SRR19364627, SRR19364628, SRR19364629, SRR19364632, SRR19364641, SRR19364644, SRR19364645, SRR19364654, SRR19364658 (Bailey et al., 2024), and Bioproject PRJEB47918 (Durward‐Akhurst et al., 2021). Data from Tozaki et al. (2021) can be accessed through the Open Science Framework (https://doi.org/10.17605/OSF.IO/PVNCY).
