# Supplementary material for: A missense mutation in the KCNE4 gene is not predictive of equine anhidrosis
Source: Anim Genet. 2025 Feb 15;56(1):e70004. doi: 10.1111/age.70004 (PMC11829550; doi:10.1111/age.70004)
Supplement: Supplementary file 1 — Figure S1. [file AGE-56-0-s002.docx]

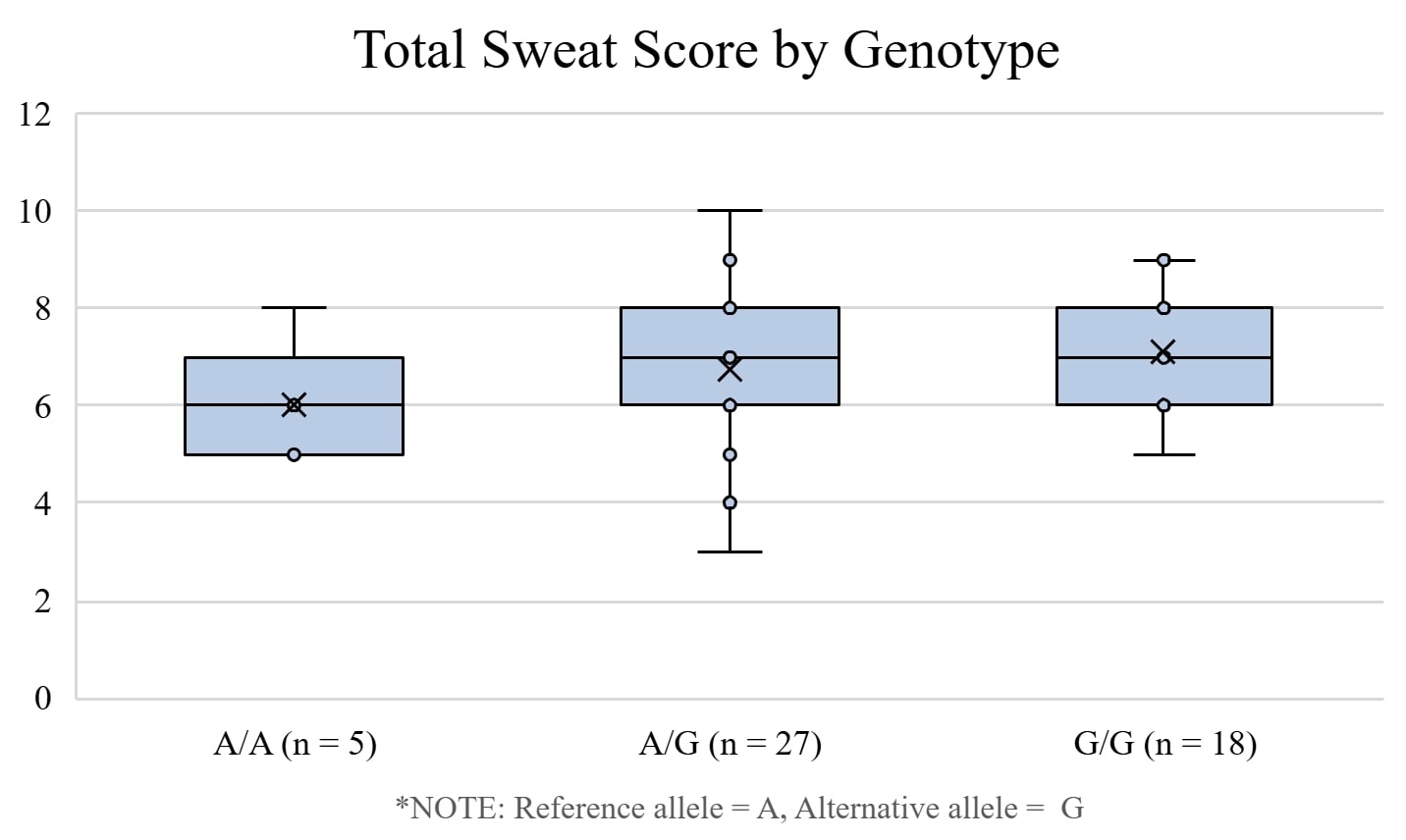


**Supplementary Figure 1.** Box-whisker plot of total sweat scores classified by horse genotype at marker rs68643109. The A allele is the reference allele.
